# A Social-Ecological Framework of Theory, Assessment, and Prevention of Suicide

**DOI:** 10.3389/fpsyg.2017.01756

**Published:** 2017-10-09

**Authors:** Robert J. Cramer, Nestor D. Kapusta

**Affiliations:** ^1^School of Community and Environmental Health Sciences, Old Dominion University, Norfolk, VA, United States; ^2^Suicide Research Group, Department for Psychoanalysis and Psychotherapy, Medical University of Vienna, Vienna, Austria

**Keywords:** suicide, prevention, social-ecological model, risk assessment

## Abstract

The juxtaposition of increasing suicide rates with continued calls for suicide prevention efforts begs for new approaches. Grounded in the Centers for Disease Control and Prevention (CDC) framework for tackling health issues, this personal views work integrates relevant suicide risk/protective factor, assessment, and intervention/prevention literatures. Based on these components of suicide risk, we articulate a Social-Ecological Suicide Prevention Model (SESPM) which provides an integration of general and population-specific risk and protective factors. We also use this multi-level perspective to provide a structured approach to understanding current theories and intervention/prevention efforts concerning suicide. Following similar multi-level prevention efforts in interpersonal violence and Human Immunodeficiency Virus (HIV) domains, we offer recommendations for social-ecologically informed suicide prevention theory, training, research, assessment, and intervention programming. Although the SESPM calls for further empirical testing, it provides a suitable backdrop for tailoring of current prevention and intervention programs to population-specific needs. Moreover, the multi-level model shows promise to move suicide risk assessment forward (e.g., development of multi-level suicide risk algorithms or structured professional judgments instruments) to overcome current limitations in the field. Finally, we articulate a set of characteristics of social-ecologically based suicide prevention programs. These include the need to address risk and protective factors with the strongest degree of empirical support at each multi-level layer, incorporate a comprehensive program evaluation strategy, and use a variety of prevention techniques across levels of prevention.

Suicide rates in the United States are increasing in the last decade from 11.0 (per 100,000) in 2004 to 13.4 in 2014 (Drapeau and McIntosh, 2014). Most recent data summarized by the Centers for Disease Control and Prevention (CDC) echoes this pattern with detailed analyses showing that trend inclines may be moderated by factors such as gender, age, and method (Centers for Disease Control and Prevention, 2016a). What is particularly concerning is that the increasing suicide rate is occurring in the presence of a 2012 national suicide prevention strategy put forth by the U.S. Surgeon General's Office, which is based on four broad strategic directions reflecting a multi-level perspective: (1) create supportive environments promoting healthy, empowered persons, families, and communities; (2) enhance community-oriented prevention services; (3) promote timely, supportive services, and; (4) improve suicide-related surveillance data (United States Surgeon General's Office, 2012).

Despite progress in the effectiveness of suicide prevention efforts (Mann et al., 2005; Zalsman et al., 2016), suicide prevention still suffers from several critical limitations: the inability to predict suicidal behavior in individuals (Fowler, 2012; Chu et al., 2015; Chan et al., 2016), inconsistent suicide-related terminology (Skegg, 2005; Silverman and De Leo, 2016), lack of multi-level theoretical development (O'Connor, 2011; Barzilay and Apter, 2014), and insufficient implementation of multi-level prevention programs (Hegerl et al., 2008; van der Feltz-Cornelis et al., 2011). Informed by theoretical, risk/protective factor, and prevention program evidence, we articulate a conceptual multi-level framework for suicide prevention.^1^ We further make recommendations concerning development of multi-level suicide risk theory, research, assessment and prevention.

## The state of suicide prevention efforts

The current scope of suicide prevention efforts spans primary prevention (e.g., public awareness campaigns), secondary prevention (e.g., gate-keeper training programs), tertiary prevention (e.g., psychotherapy), and postvention (e.g., survivor support groups). Extending these traditional categories on a mental health intervention spectrum, suicide prevention can be applied at a universal (i.e., to the general public), selective (e.g., groups defined by lifetime risk such as military personnel), and indicated prevention (i.e., high risk groups where risk is already elevated—e.g., psychiatric inpatients) levels (Institute of Medicine Committee on Prevention of Mental Disorders., 1994). A recent systematic review summarizes the overall state of effective suicide prevention programs across these levels (Zalsman et al., 2016). The findings support reduction of suicide-related thoughts and behaviors (i.e., ideation, attempts and completed suicide) for: (1) restricting access to lethal means (e.g., hot-spots for jumping), (2) school-based awareness programs, (3) lithium and clozapine use, and (4) psychotherapeutic efforts for depression. Authors also noted a lack of current evidence for an array of other prevention approaches (e.g., gatekeeper training, physician and public education).

Of the few approaches that cut across more than one of multiple potential levels of prevention, recent efforts by the United States (US) National Action Alliance for Suicide Prevention have focused on initiatives (e.g., Zero Suicide, Vision Zero) toward the goal of absolute elimination of suicide (Erlich, 2016). Components of these approaches range from improving follow-up practices with patients post-discharge and maintaining contact with at-risk persons to enhancing infrastructure (e.g., personnel, training content) and prevention resources. Two promising tertiary suicide prevention strategies common to clinical psychiatry are Dialectical Behavior Therapy (DBT) (Comtois and Linehan, 2006) and the Collaborative Assessment and Management of Suicide (CAMS) (Ellis et al., 2015). A noteworthy gap is that, even clinical or targeted approaches showing potential effectiveness are unable to simultaneously target the individual through societal level influences on suicide risk.

## A multi-level understanding of suicide prevention

We echo other calls in the literature for a multi-level public health approach to suicide prevention (Dahlberg and Krug, 2002; van der Feltz-Cornelis et al., 2011). The CDC provides valuable guidance based on the assumption that prevention efforts for any health or disease issue require integrated multi-level efforts within a Social-Ecological Model (SEM) (Centers for Disease Control Prevention, 2017). The SEM is a four tier framework for organizing risk and protective factors, which then inform corresponding prevention strategies. From macro to micro levels, the four strata are: societal, community, relational, and individual levels. Societal factors concern larger scale issues such as social and cultural norms, policies, and other guiding rules or laws. Community level influences are those circumscribed to a certain region like neighborhood centers, schools, workplaces and healthcare providers. Relational factors are those defined by direct person-to-person interaction such as social support or withdrawal, peers, and family. Individual level factors pertain to person characteristics such as demographics, attitudes, health conditions, and others. The SEM has been meaningfully applied to a range of health issues and prevention programs such as health literacy (McCormack et al., 2017) and vaccine usage (Kumar et al., 2009).

We see at least three straightforward benefits of such a multi-level schema. First, suicide risk and protective factor literature tends to be fragmented by SEM level. That is, even where summaries of risk factors are provided, they are often limited to one or two SEM levels. An SEM of suicide prevention, therefore, provides a potentially comprehensive framework for organizing risk and protective factor knowledge; as such, it is a working template for adding new factors, as well as integrating levels to examine how upper level factors may moderate the influence of lower level factors, and vice versa.

Following from enhanced organization of factors, a second benefit is that an SEM of suicide prevention can provide grounding for multi-level intervention and prevention program design and implementation. This idea has been demonstrated by closely-related comprehensive approaches to prevention of gun violence prevention (Rubens and Shehadeh, 2014) and campus sexual assaults (Centers for Disease Control Prevention, 2016b). For instance, Rubens and Shehadeh organized potential interventions and preventions for gun violence in the US along levels of the SEM, noting potential strategies ranging from individual (e.g., parent-child relationships) to societal (e.g., financial liability for those violating gun safety norms) approaches (Rubens and Shehadeh, 2014). Finally, articulation of a multi-level approach to suicide prevention can provide a framework for the re-organization of current theories of suicide. That is, to date causal theories of suicide consistently fail to fully integrate multi-level perspectives. It is our hope that a social-ecological view of suicide prevention would spur growth and effectiveness in theory and practice.

## Toward a social-ecological model of suicide prevention

In support of a multi-level approach to suicide prevention, Caine proposed to frame suicide prevention within an SEM model in terms of its shared risk with interpersonal violence (Caine, 2013). However, the resulting ecological model of shared risk was limited in scope in terms of merely listing sample risk and protective factors in common for both suicide and interpersonal violence. Extending this approach we articulate a comprehensive picture of risk and protective factors associated with at least one aspect of suicide-related thoughts and behavior, yielding the SESPM.

### Search strategy and selection criteria

^2^ In order to balance comprehensiveness of sources cited, while also recognizing brevity of this manuscript format, we did the following to identify sources to inform the SESPM. We searched Pubmed, Medline, Psychinfo, and Psycharticles using combinations of the following phrases: “suicide,” “risk factor,” “protective factor,” “prevention,” “intervention,” “review,” and “meta-analysis” while focusing on articles from 1980 to present. Reviews and meta-analyses were given priority because we aimed to provide a big picture review (see Table 1). We further used Google Scholar to identify pertinent content from the following major professional organizations: American Foundation for Suicide Prevention, Suicide Prevention Resources Center, American Association of Suicidology, World Health Organization, CDC, and Substance Abuse and Mental Health Services Administration. Once a full set of key sources was identified, we dropped sources that were completely redundant with others.

**Table 1:** Compilation of major suicide risk and protective factors organized by levels of centers for disease control and prevention's social-ecological model.

**Risk factors**	**Protective factors**
Societal: Economic downturn/depression Living location with less restrictive firearm laws Seasonal variation Stigma about mental health and treatment Air pollutants Viruses/parasites Poverty Mountain region of the US Western and southern US	Societal: Healthy economy Living location with more restrictive firearm laws Mental health funding Northeast US
Community: Exposure to community violence ***Local suicide epidemic*** Barriers to healthcare access	Community: Crisis support lines/hotlines Healthcare/mental healthcare access Effective mental healthcare Trained gate keepers Community involvement School-based support and intervention programming^*^
Interpersonal/Relationship: Living in household with firearm ***Exposure to suicide/contagion*** Family violence Family conflict Family history of mental illness Family history of suicide/attempt Relationship instability Death of a loved one Severing of romantic relationship Social isolation/withdrawal Combat exposure^*^	Interpersonal/Relationship: ***Presence of social support*** ***Use of social support*** ***Perceived social support*** Concerns suicide is harmful to child/family Sense of responsibility to family Healthy long-term committed relationship/marriage Help-seeking behavior Children present in the home Pregnancy^*^ Pulling together Caring letters Social connectedness Contact with caregivers^*^ Support for connection with healthcare providers Cognitive-behavioral therapy Dialectical-behavior therapy Collaborative assessment and management of suicide (CAMS)
Individual:	Individual:
Biological Male sex (completions)/Female sex (attempts)^*^ Serotonin dysfunction Family history of suicidal behavior	Biological SSRI usage ***Lithium/mood stabilizer treatment*** ***Clozapine usage***
Socio-Demographic Gender (e.g., Transgender status) Lesbian, gay, bisexual or other sexual orientation minority identity^*^ Religiosity/spirituality (i.e., suicide as a resolution to problems)^*^ Native American ethnicity^*^ Hispanic ethnicity^*^ Asian/Pacific Islander ethnicity^*^ Whites (compared to non-Whites)^*^ Older adult age^*^ Middle adult age^*^ High risk professions (e.g., military, law enforcement)^*^ Firearm ownership (and unlocked, loaded)^*^ Incarceration^*^ High perceived/subjective stress Job loss/unemployment Financial strain Recent discharge from psychiatric hospital^*^ Bullying/bias crime victimization^*^	Socio-Demographic Heterosexual sexual orientation Religiosity/spirituality (i.e., beliefs about suicide being wrong)^*^
Psychiatric ***Mental health diagnoses/symptoms such as depression, bipolar***, post-traumatic stress disorder, anxiety, and active phase schizophrenia ***Personality disorders such as Borderline Personality*** ***Substance use/abuse (e.g., cannabis)*** ***Alcohol use/abuse***	Psychiatric Treatment motivation
Psychological ***Prior suicide attempt*** ***Current suicidal thinking*** ***Presence of suicidal intent*** ***Presence of suicide plan*** ***Access to/presence of lethal means*** ***Preparatory behaviors (e.g., giving away prized possessions)*** Prior or current non-suicidal self-injury History of other suicide (e.g., ideation) ***Hopelessness*** Low self-control/high impulsivity Aggression Agitation Emotion dysregulation Severe mood change Childhood abuse ***Feelings of burdensomeness*** ***Rejection/thwarted belonging*** Chronic illness^*^ Acute health symptoms^*^ Fatigue Sleep disturbance/disorders Neuroticism Introversion Limited openness to experience Perfectionism Homelessness^*^ Low self-esteem Shame Physical pain tolerance Fearlessness of suicide/death Thinking errors/negative thinking Psychache/psychic pain Internalized stigma^*^	Psychological ***Coping skills*** Problem solving skills Moral objections to suicide Survival beliefs/desire to live Fear of suicide/death Fear of social disapproval Optimism ***Hopefulness/positive future orientation*** Life satisfaction Intact reality testing High self-esteem/self-efficacy Resiliency Extraversion ***Additional reasons for living***

* *Risk or protective factor demonstrating unique importance for a specific population*.

***Bold italics font**,strongest risk/protective factor for suicide risk*.

Selection of the final integrated body of existing evidence represented scoping/conceptual summaries (Bryan and Rudd, 2006; Van Orden et al., 2010; Drapeau and McIntosh, 2014; Bernard et al., 2015), systematic reviews/meta-analyses (Serafini et al., 2012; Calear et al., 2016; Chan et al., 2016; Ma et al., 2016; Zalsman et al., 2016; Franklin et al., 2017), mortality risk studies (Björksenstam et al., 2015, 2016; Madsen et al., 2017), measure development (Linehan et al., 1983), and policy analysis (Anestis and Anestis, 2015) into a unified SESPM framework, as presented in Table 1. In doing so, we differentiate factors widely applicable across groups vs. those that tend to demonstrate population-specific associations with suicide (e.g., military veterans, youth, lesbian, gay, bisexual and transgender [LGBT] persons). For example, concerning LGBT youth, literature consistently links population-specific experiences of internalized stigma and victimization as associated with suicide risk; moreover, sexual orientation minority status itself is linked with elevated suicide risk (Haas et al., 2011; Duncan and Hatzenbuehler, 2014). The need for attention to nuance of even strong risk factors varying by population is further illustrated by primary psychiatric diagnoses linked to suicide. For instance, standardized mortality risk (SMR) and other research documents the exacerbated prominence of depression, bipolar, and cluster B personality disorders (e.g., borderline, antisocial) in enhancing risk for death by suicide among psychiatric patients (Björksenstam et al., 2015, 2016; Madsen et al., 2017). To illustrate, cluster B disorders are associated with SMRs in this population as high as 33–34 (Björksenstam et al., 2015; Madsen et al., 2017). Cannabis use and dependence, another diagnostically relevant disorder category, has been shown to be associated with exacerbated suicide risk, especially among adolescents and particularly when the cannabis is associated with experiencing of other psychiatric conditions (e.g., psychosis) (Serafini et al., 2012). Thus, cannabis use or dependence may also serve as poor coping or a pathway to suicide among adolescent youth.

We also note many factors with the strongest, most consistent associations with suicide risk (see Table 1), defining strongest and consistent in terms of effect sizes and odds ratios related to suicide-related thoughts or behaviors, direct associations with suicide (e.g., serving as a mediator), as well as those that are highlighted by clinical and prevention experts as those requiring attention across populations (e.g., depression). In all, SEM levels with the strongest support tend to be individual and interpersonal/relational levels. For instance, at the individual or interpersonal levels we note risk factors with the strongest associations with suicide-related thoughts and behavior such as a prior suicide attempt, diagnosis of depression or bipolar disorders, and suicide contagion. To illustrate, hopelessness provides a clear example of an individual psychological risk factor with considerable support; hopelessness has been identified as an independent risk factor for suicide requiring clinical assessment (Bryan and Rudd, 2006), and empirical data raises the potential that hopelessness may serve as a pathway to suicide-related thoughts and behavior explaining the influence of other risk factors (e.g., thinking styles) (Abramson et al., 1998). Moreover, protective factors such as presence, use and perception of positive social support is denoted as among the strongest factors (see Table 1 for full list of demarcated factors with strongest research support). As such, from a public health education standpoint, the integrated summary may serve to reinforce the key factors to include in dissemination efforts by public organizations.

Given the fact that suicidology is an ongoing research field, the proposed SESPM is not intended to be exhaustive. The purpose of the SESPM is a guide to move research and prevention forward, as well as to provide a framework for understanding nuance in suicide prevention. To illustrate the latter point, examination of the multi-level organization identifies several levels at which for example firearm-related factors may influence suicide risk. While firearm access or ownership is associated with elevated suicide risk (Anglemyer et al., 2014), this link may be moderated by other individual (e.g., safe storage) (McCarten et al., 2001), relational (e.g., restricted means counseling) (Stanley et al., 2016), and societal (e.g., firearm restriction laws) (Anestis and Anestis, 2015) factors. The SESPM provides a summary of literature in order to build toward better mediation, moderation, and causal research, as well as multi-level prevention efforts.

## Recommendations for SESPM-informed research, theory and programming

Consequently, we advocate five next steps for the suicidology field.

### Empirical testing and adaptation

**Figure 1:**
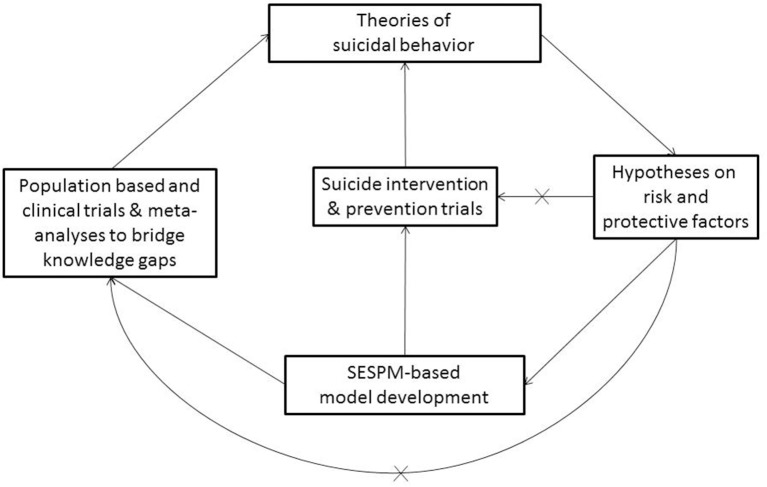
Conceptual SESPM Model for theory, assessment and prevention program development. X, pathways in current suicide research that should be avoided in SEPSM-based model development.

We adopt the view that the SESPM is both preliminary and fluid, suggesting prevention may need to account for population- or context-specific considerations. For instance, the SESPM itself may vary by population or culture. To illustrate, it is well known that risk factors such as Human Immunodeficiency Virus (HIV) status itself (Carrico et al., 2007) and internalized HIV-related stigma (Cramer et al., 2015) play particularly salient roles in suicide risk, whereas other factors may be less important for this group. Additionally, the SESPM offers a clear organizational approach to future systematic reviews and hierarchical approaches to meta-analysis or regression. In all, with future empirical testing, the SESPM may need refinement or adaptation by population over time. We advocate in a first step to conduct risk and protective factor meta-analyses to develop appropriate SESPM templates for risk groups. In a second step, this quantitative information about the weight of risk factors should be used in population trials consequently (see Figure 1).

### A framework for public health education and training efforts

Promising suicide prevention education and training programming exists for the public (Teo et al., 2016) and medical/health professionals (Cramer et al., 2017), yet these areas are in need of further study (Zalsman et al., 2016). Moreover, empirically-tested educational prevention strategies often lack consistent structural framing. That is, content and modalities of these trainings vary, often neglecting content such as community and societal level risk factors and prevention efforts. As has been done in the development of other public health prevention approaches such as HIV prevention (Baral et al., 2013), we encourage development of research/data summaries, educational materials, and training content to be organized by SESPM levels. For example, graduate training or continuing education programs may address established suicide prevention-related competencies (Rudd et al., 2008; Cramer et al., 2013) by SESPM level. While a full program-wide review of such competencies is beyond the scope of this piece, using structured training approaches like observed structured clinical examinations (OSCEs), health professions literature (Hung et al., 2012; Cramer et al., 2016) highlights necessary skills for health providers like knowing empirically-indicated risk/protective factors and intervention/support possibilities. Established training models such as patient simulation or online-mediated courses can integrate such skill development and factual content into a SESPM framework. The end goal of such an approach would be that SESPM-educated health clinicians may be able to make better use of their multi-level knowledge and skills in working with at-risk individuals or designing stronger prevention programs.

### Multi-level suicide risk theory

Historically, public health and health science prevention efforts have lacked adequate theoretical grounding. Recent health professions literature argues that effective prevention efforts requires strong grounding to bolster effective health behavior change (Im, 2015; Prestwich et al., 2015; Krieger, 2016). The advantages of theory-informed public health include conceptualization of multi-level prevention/intervention programming, transdisciplinary communication, and accounting for practical societal and scientific influences (e.g., funding, political issues). Interestingly, social and behavioral science literature focuses on suicide as the subject of theoretical speculation, although there are varying levels of empirical testing and support across these theories.

Although a full review of all contemporary suicide risk theories is beyond the scope of this work, we provide an example theory with short description for each SESPM level for illustrative purposes (see Table 2). From top down, example theories can be seen in societal (e.g., *Le Suicide*) (Durkheim, 1897), community (e.g., Military Transition Theory) (Castro and Kintzle, 2014), relational (e.g., Interpersonal-Psychological Theory of Suicide) (Joiner, 2005; Van Orden et al., 2010), and individual (e.g., Cubic Model of Suicide) (Shneidman, 1981) level perspectives. In his seminal text *Le Suicide*, sociologist Emil Durkheim theorized suicide as a reaction to the intersection of social integration (the clustering of people in social groups) and regulation (the extent of rituals and customs being influenced by societal norms) (Lester, 1999). Rooted in a litany of causes for suicide among US veterans, Military Transition Theory highlights suicide risk as a function of factors unique to the community of military personnel reintegrating into civilian life (Castro and Kintzle, 2014). A three-stage transition is posited: approaching, managing, and assessing the transition. These stages imply a degree of multi-level influence in that they require the person to navigate and evaluate individual, familial, work and other challenges.

**Table 2:** Sample suicide prevention and intervention strategies by level of the social-ecological model.

**Social-ecological model level**	**Sample intervention and prevention programming**	**Sample theory of suicide**
Societal	1. Firearm laws or regulations concerning storage, mental health background checks, etc.	Sociological theory of suicide
	2. Public awareness campaign targeting mental health and therapy stigma reduction.	
	3. Suicide-specific federal funding initiatives.	
Community	1. Crisis support lines.	Military transition theory
	2. Free mental health screenings provided by community mental health centers or in clinics treating high risk populations.	
	3. School-based programs targeting diversity-related social norms, mental health care access, or suicide awareness.	
Relational	1. Group psychotherapy.	Interpersonal-psychological theory of suicide
	2. Individual psychotherapy.	
	3. Gate keeper training.	
Individual	1. Adoption of positive health behaviors (e.g., exercise, food choices, sleep hygiene)	Cubic model of suicide
	2. Mental health literacy courses.	
	3. Positive coping skills training/adoption.	

The Interpersonal-Psychological Theory of Suicide (IPTS) posits that suicide thinking is a function of self-perceptions in relation to others; negative interpersonal cognitions occur in two forms: thwarted belonging and perceived burdensomeness (Van Orden et al., 2010). Ideation transitions to an attempt when the individual has developed sufficient habitation to pain and fearlessness of death in order to commit the act. In this way, the IPTS may be considered both individual and relational in nature. Finally, Shneidman articulated an individual theory of impulsive suicide (Shneidman, 1981). The impulsive act, calculated to be fatal by the attempter, is thought to occur in the presence of acute psychological states of stress, agitation and psychache (i.e., emotional pain).

Relying on the SESPM organizational framework of risk and protective factors (see Table 1), we believe a valuable next step in the theoretical development and testing is a *multi-level or social-ecological theory of suicide* in both population-based and clinical trials, as well as in interventions (see Figure 1) in order to inform theory development and prevention programming. The “x” lines refer to the idea that suicide research suffers from the problem of repeated over simplification of studies bypassing a comprehensive use or development of a multi-level model. Therefore, as illustrated in Figure 1, it is critical to understand that the proposed SESPM, a multi-level organizational framework, does not constitute a true theory of suicide by itself. However, following the pathways outlined in the Figure, a consistent and causal theory of suicidal behavior should be deductible from such a framework, and therefore be empirically testable (Horvath, 2016). A social-ecological theory of suicide, for instance, may specify individual attitudes, traits and mental health symptoms as primary, direct predictors of imminent suicide risk. Complementing that testable hypotheses, societal or community level factors, as well as sub-population variation, may serve as directly influence chronic risk, while also playing moderating roles concerning how individual and relational factors affect imminent or acute risk.

### Enhancing suicide risk assessment methods

Despite a proliferation of suicide risk assessment tools (Lotito and Cook, 2015), recent evidence suggests limited ability to predict future suicidal behavior (Chan et al., 2016; Large et al., 2016). Self-report instruments suffer an identical limitation much of suicide-related theory and risk/protective factor summaries have: a lack of accounting multi-level understanding. For example, the most commonly used self-report tools often assess the frequency and nature of past/present/future ideation and attempts, strongly correlated mental health symptoms (e.g., depression, hopelessness), or protective factors (e.g., reasons for living). While such information is clinically useful to fill in gaps not otherwise captured in interview (Lotito and Cook, 2015), it is still limited in scope.

Several assessment structures exist in the literature, sometimes hinting at the need to address multi-level facets. Such attempts have led to different recommendations for the development of suicide risk assessment tools, but none of these strategies have seen effective implementation yet. For example, suicide risk assessment is often based on lists of symptoms without an integrated perspective (Kral and Sakinofsky, 1994), therefore proposing a model that comprises both background and subjective suicide risk factors. The former are the socio-demographic indices associated with increased risk which are based on different populations and cultures, and are prone to change over time. The latter, background risk factors, can inform the clinician about a patient's general level of risk, while the assessment of individual factors focusses on emotions, cognition, idiosyncratic meanings, general mental state, and experience. Suicide risk assessment methods can also be based on factors falling into (1) individual, (2) clinical, (3) interpersonal, (4) situational, and (5) demographic categories, thus encompassing some of the SESPM levels suggested herein (Simon, 2011).

In total, the variation in recommended methods suggests that a one-size-fits-all solution to suicide risk estimation is an ill fit. Such methodological complexities might be responsible for the result of the most recent meta-analysis of suicide risk assessment scales, which concluded that there is insufficient evidence to support the use of risk scales and tools in clinical practice due to the rather low positive predictive value (PPV) of the scales, which ranged between only 1.3 and 16.7% (Chan et al., 2016), with 87% false positives, a clinically imprecise, economically intensive and unnecessarily stigmatizing proportion. We argue that the heterogeneity and confusion about suicide risk assessment methods has its primary origin in the lack of a unified and empirically testable theory of suicidal behavior.

We posit that the next meaningful steps in suicide risk assessment tool development may lie in two areas: (1) a psychometrically-validated structured professional judgment (SPJ) of key multi-level risk factors, such as the Screening Tool for Assessing Risk of Suicide (STARS) protocol (Hawgood and De Leo, 2016) (which does not fully account for multi-level influences) and (2) a multi-level suicide risk assessment algorithm. For example, although a potentially time, resource and funding intense project, we recommend development of a suicide risk assessment tool for use by mental and medical health professionals that addresses risk and protective factors across all four layers of the SESPM. Such a new approach may be translated into a SPJ tool, an approach to mental health assessment that provides semi-structured rating forms to be used by trained health professionals. In addition, post-interview, more rigorous interviewer-rated checklists, could help to refine and validate the SPJ tool. Nowadays, online implementation and translation in different languages allows for the development of a globally available instrument for suicide risk assessment within an SESPM model for ongoing refinement. Violence risk literature provides examples of well-validated SPJs accounting for three SEM levels, accounting for empirically-indicated risk and protective factors for interpersonal violence, including individual (e.g., affective stability, substance use), relational (e.g., treatment compliance, personal/social support), and community (e.g., living situation, professional services) level issues (Douglas et al., 2013). Community level risk is further accounted for by the relevance of these factors to the setting of evaluation (e.g., inpatient hospital vs. outpatient clinic). Using the SESPM, it is plausible that a suicide SPJ could be developed by first identifying and testing a lengthy set of risk of factors, exposing the preliminary instrument testing in emergency room, outpatient clinic and inpatient hospital settings. After initial reduction and psychometric evaluation of the instrument, further testing would be required for longitudinal and cross-cultural validation. Likely, a culturally-adapted version of such an instrument would be quite useful.

Alternatively, it may be beneficial to develop a risk assessment algorithm, which is stratified by available knowledge (i.e., different sets of risk factors might be relevant for males vs. females, adolescents vs. elderly, etc.). As such, a computer-based suicide risk estimation algorithm may be developed in which a clinician can collect comprehensive multi-level patient and situational information, entering the information into a weighted equation. The first step in such effort is mathematical identification of a weighted formula, likely based on a validated multi-level theory of suicide that has to be developed. First approaches in the direction of a suicide risk algorithm have been provided; however, the presented tool was limited to six individual level factors (e.g., age, self-harm history) suitable for depressed persons for the prediction of suicidal ideation only (Liu et al., 2016). With simultaneous systematic review of multi-level risk and protective factors for suicide ideation, attempts, and completed suicide, identification of relative weights for an SESPM-based algorithm based suicide risk assessment tool should be attainable.

### Development of multi-level prevention programming

First and foremost, SESPM-based research is needed to inform best practices for prevention programming. Thus, our recommendations for development of multi-level programming are provided with the caveat that further research and theory development are required. As a starting point, multi-level suicide prevention should address (1) general practitioner education concerning depression and suicide; (2) increased access to care for high-risk groups, and; (3) emphasis on restricting access to lethal means (van der Feltz-Cornelis et al., 2011). While we agree with the importance of these practical recommendations, we further posit that design of an ideal multi-level approach to suicide prevention would possess the following characteristics: (1) incorporation of the risk and protective factors with strongest empirical support relative to the population (e.g., general population vs. high risk psychiatric inpatients); (2) use of prevention strategies at each SESPM level; (3) inclusion of a multi-level program evaluation strategy including data gathered from patients and other stakeholders (e.g., therapists, policy makers, etc.)—patient data would include suicide and self-harm, whereas additional patient and stakeholder information could cover subjective and objective patient-oriented outcomes more generally; (4) grounding in relevant theory to inform mechanisms of change; and (5) presence of prevention efforts using at least primary and secondary prevention techniques where possible.

## Limitations and conclusions

The SESPM perspective holds a number of limitations warranting attention. For example, one shortcoming of the piece is that the present summary of factors did not rise to the level of rigor as formal systematic reviews (nor was it our intention to do so). Moreover, the SESPM is not exhaustive; rather it is intended to provide a flexible framework for additional research and program development moving forward. Another limitation of the present discussion can be seen in failing to conduct meta-regression or analysis; future scholarship may offer very important confirmation or modification of the framework via such analyses. A notable limitation of our SESPM-based recommendations is the labor, time and resources necessary for education and training efforts; however, we argue that the cumbersome processes involved in many of the SESPM-based recommendations is ultimately worthwhile in the long run because multi-level suicide prevention efforts may save lives and improve quality of life beyond what is currently within the capabilities of public health and clinical mental health fields. Also, while the same limitations concerning time, cost and resources certainly apply to risk assessment tool development recommendations, we believe that the scientific progress in suicide prevention is not a question of experienced clinical rating vs. algorithm building, but both approaches mutually informing each other to create new insight.

We have articulated background, structure and recommendations for a SESPM. The bottom line of our perspective is that we agree with a sentiment that has been expressed in prior suicidology literature: scholars and practitioners alike need to expand how we think about suicide. The SESPM represents a valuable step in moving from a hyper-focus on individual-level suicide risk prediction toward a comprehensive multi-level perspective on suicide prevention. We welcome further dialogue, research and development moving forward.

## Author contributions

Both authors contributed equally to this paper in performing literature search, conceptualizing, drafting, and revising the manuscript.

### Conflict of interest statement

The authors declare that the research was conducted in the absence of any commercial or financial relationships that could be construed as a potential conflict of interest.

## Footnotes

^1^Authors recognize not every construct can be defined due to the brevity of personal views format articles.

^2^Our paper is not intended to be a systematic review; therefore, full search criteria and other details are not reported.
